# Integrative RNA-Seq Analysis Reveals Stress Type-Dependent lncRNA-Centered Co-Expression Networks Across Human Cellular Stress Contexts

**DOI:** 10.3390/ijms27167288

**Published:** 2026-08-15

**Authors:** Christina Anastasiadi, Aggeliki Kasapi, Ioannis Sentementes, Vasileios Gouzouasis, Margaritis Tsifintaris, Antonis Giannakakis

**Affiliations:** 1 Department of Molecular Biology and Genetics, Democritus University of Thrace, 68100 Alexandroupolis, Greece; anastasiadichristine@gmail.com (C.A.); aggelikikasapi@gmail.com (A.K.); giannosentementes@gmail.com (I.S.); vgouzouasis@gmail.com (V.G.); 2 Computational Medicine Center, Thomas Jefferson University, Philadelphia, PA 19017, USA; 3 University Research Institute of Maternal and Child Health and Precision Medicine, National and Kapodistrian University of Athens, 11527 Athens, Greece

**Keywords:** lncRNA, long non-coding RNA, cellular stress response, systems biology, oxidative stress, hypoxia, heat stress, UV-induced DNA damage, RNA-seq, WGCNA, ASTRA

## Abstract

Long non-coding RNAs (lncRNAs) are emerging as important regulators of cellular adaptation to environmental and molecular stress, but the extent to which their responses remain reproducible and stress-type-dependent across human stress conditions remains unclear. Here, we performed an integrative transcriptomic meta-analysis of human stress-response datasets from ASTRA and GEO, focusing on normal, non-cancerous, wild-type human cell lines exposed to oxidative stress (H_2_O_2_), hypoxia, heat stress, or UV-induced DNA damage. Gene Ontology (GO) enrichment analysis of differentially expressed (DE) protein-coding mRNAs confirmed that the resulting stress-stratified comparison captured biologically coherent transcriptional programmes to oxidative stress signaling, hypoxic and metabolic adaptation, heat-induced proteostasis, UV-induced DNA damage signaling, apoptosis, and cell-cycle regulation. Differential expression analysis was subsequently integrated with Weighted Gene Co-expression Network Analysis (WGCNA) -based module–treatment associations to rank network-supported lncRNA candidates. By prioritizing candidates for recurrence across at least two different stress studies, we identified lncRNAs with increased recurrence and consistent stress-type-dependent expression regulation, embedded within coordinated stress-associated mRNA programs.

## 1. Introduction

Long non-coding RNAs (lncRNAs) are no longer regarded as transcriptional by-products or “non-coding noise”, but as dynamic architects of cellular states [1,2]. Through their interactions with DNA, RNA, proteins, chromatin-modifying complexes, and transcriptional machinery, lncRNAs contribute to the organization of gene-regulatory networks that define cellular identity, plasticity, and adaptation [3,4,5]. Rather than acting through a single universal mechanism, lncRNAs operate across multiple regulatory layers, including chromatin remodeling, transcriptional control [4], post-transcriptional regulation, RNA stability, and molecular scaffolding [6,7]. This functional versatility positions lncRNAs at the interface between environmental cues and cellular decision-making, enabling them to modulate how cells respond to developmental signals, metabolic perturbations, and stress-induced challenges [5,8].

Beyond their regulatory functions, lncRNAs are increasingly recognized as promising functional biomarkers with potential clinical relevance [3,9]. Their tissue-, cell-type-, and condition-specific expression patterns make them particularly attractive for monitoring dynamic biological states that may not be captured by conventional protein-coding markers alone [1,3,9]. Importantly, because many lncRNAs are not merely passive indicators of disease or stress but active components of regulatory circuits, they may serve a dual role as both biomarkers and mechanistic entry points for intervention [3,10]. In this context, lncRNAs hold promise as future personalized therapeutic targets in functional medicine, where molecular signatures could be used not only to classify disease states or stress-response phenotypes, but also to guide individualized strategies aimed at restoring cellular homeostasis [3,10].

Molecular stress represents a fundamental biological condition in which cells are exposed to internal or external perturbations that challenge homeostasis [11]. During evolution, the ability to sense, interpret, and respond to stress has been essential for cellular survival, organismal adaptation, and the emergence of complex regulatory systems [12,13]. Rather than being merely harmful, stress can act as a selective and instructive force, shaping adaptive mechanisms that enable cells to remodel metabolism, repair damage, alter proliferation, activate defense programs, or enter dormancy, senescence, or death [11]. At the molecular level, stress responses are largely mediated by rapid, coordinated changes in gene expression, enabling cells to translate environmental or intracellular signals into transcriptional [14] and post-transcriptional [15] programs that determine cell fate [11,12]. However, the consistency of these stress responses has not been systematically studied across different cellular backgrounds.

Different types of molecular stress activate partially distinct but potentially interconnected response pathways [16,17]. Oxidative stress arises from an imbalance between reactive oxygen species production and antioxidant defenses, leading to damage of proteins, lipids, DNA, and mitochondria [18,19]. Hypoxia reflects reduced oxygen availability and drives metabolic adaptation, angiogenic signaling, and HIF-dependent transcriptional remodeling [20]. Heat stress primarily disrupts protein folding and proteostasis, activating heat-shock proteins and the unfolded protein response [21]. UV exposure and DNA-damaging stressors induce genotoxic stress, triggering DNA repair, cell-cycle arrest, apoptosis, or senescence, depending on the severity of damage and the type of cellular context [11,22]. Although these stress modalities differ in their initiating signals, they can converge on the integrated stress response [23] and related stress-responsive signaling networks, which couple proteostasis defects and other cellular perturbations, such as DNA damage, to translational control and downstream transcriptional reprogramming [12,24]. This enables cells to coordinate acute adaptation, restore proteome homeostasis, and repair molecular damage, whereas persistent or maladaptive activation may contribute to chronic dysfunction and pathological states [17,23].

Mapping the genome-wide transcriptional dynamics that associate with transient and chronic biological stress responses is therefore critical for interpreting both physiology and disease [11,14]. Acute stress-associated transcription is required for tissue repair, immune defense, metabolic flexibility, and adaptation to fluctuating environments [14,24,25]. In contrast, chronic or unresolved stress can destabilize cellular identity via transcriptional deregulation and contribute to a broad spectrum of non-inherited human diseases, including cancer, metabolic disorders, inflammatory diseases, neurodegeneration, cardiovascular dysfunction, reproductive complications, and aging-related pathologies [19,23]. Since lncRNAs are highly responsive to cell state, environmental context, and chromatin remodeling, they are well positioned to link molecular stress sensing to gene-regulatory adaptation [1,3,4]. This reciprocal relationship suggests that lncRNAs may function not only as readouts of specific molecular stressors but also as stress type-dependent regulatory components of cellular states [1,26].

Several lncRNAs have already been implicated in cellular stress responses, with well-studied examples such as *MALAT1* [26,27], *NEAT1* [28], *NORAD* [1,7], and other stress-responsive transcripts, which are associated with hypoxia, DNA damage, oxidative stress, inflammation, and cancer-related adaptation, such as *KDM7A-DT* [29]. However, it remains unclear whether lncRNAs and, more generally, the global transcriptional response is consistently deregulated in a stress type-dependent manner or predominantly defined by developmental, cell type and pathological conditions, or whether a subset of mRNAs and lncRNAs contributes to broader core-stress regulatory programs shared across molecular insults [14,16,30]. This represents an important gap, as the distinction between context-dependent and conserved stress-associated transcriptional responses may help identify novel biomarkers of stress-induced cellular adaptation and development [14,31].

Systematic investigation of transcriptional stress responses requires harmonized transcriptomic datasets with consistent annotation of stress type, treatment conditions, cell context, and transcript biotype [32]. Resources such as ASTRA [33] provide a structured framework for integrating stress-response transcriptomic datasets and enabling comparative analyses across stress modalities. This study employed ASTRA in conjunction with publicly available GEO datasets to conduct a stress-stratified, lncRNA-focused transcriptomic analysis of human cellular stress responses. The central hypothesis posits that differential expression consistency, defined as recurrence and direction across diverse cell types, could serve as an indicator of a stress type-dependent regulation of gene expression. By integrating differential expression, co-expression network, and functional enrichment analyses, we conducted a comparative genome-wide assessment of lncRNA and mRNA stress-associated responses with respect to consistency and network association. Using mRNAs as a reference and standard group, this framework enables the identification and prioritization of stress type-dependent lncRNA candidates within biologically relevant stress-associated mRNA networks.

## 2. Results

### 2.1. Integrated Analytical Workflow for Stress Type-Dependent lncRNA Prioritization

**Figure 1:**
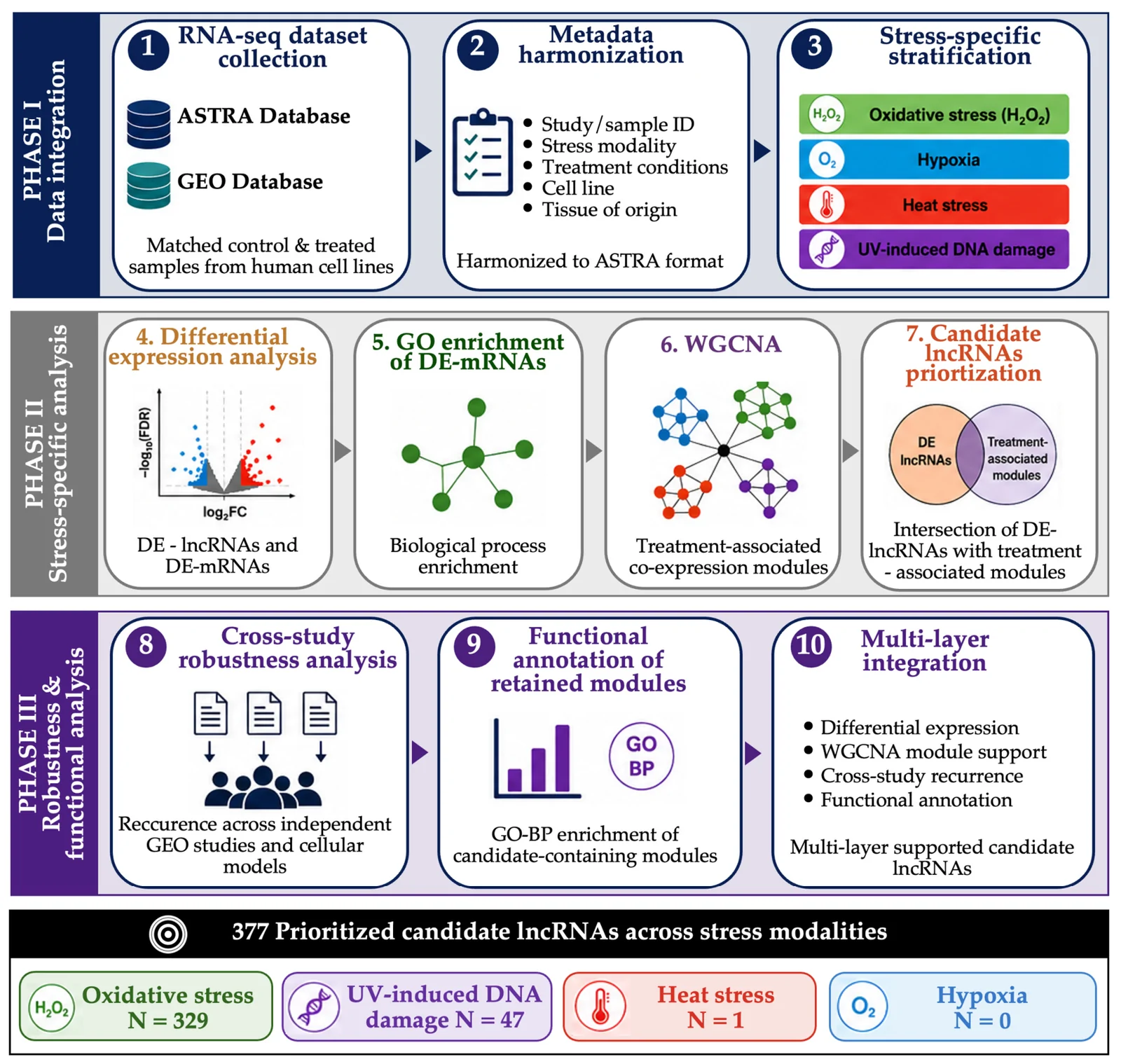
Integrated analytical workflow for the identification and prioritization of stress-associated candidate lncRNAs. RNA-seq datasets were collected from ASTRA and GEO, followed by metadata harmonization and stress type-dependent stratification into oxidative stress (H_2_O_2_), hypoxia, heat stress, and UV-induced DNA damage. Differential expression analysis and Gene Ontology (GO) enrichment of DE protein-coding genes were performed independently for each stress modality. In parallel, WGCNA identified treatment-associated co-expression modules. DE lncRNAs were integrated with treatment-associated modules to define network-supported candidate lncRNAs, which were prioritized based on differential expression magnitude, module membership (|kME|), and module–treatment correlation (|r|). Prioritized candidates were subsequently evaluated for recurrence across independent studies and cellular models, and the retained candidate-containing modules were functionally annotated using GO Biological Process enrichment analysis.

An integrated multi-layer analytical workflow was implemented to identify and prioritize stress-associated candidate lncRNAs across heterogeneous human RNA-seq datasets (Figure 1). Following RNA-seq data collection from ASTRA and GEO, metadata were harmonized, and samples were stratified into four molecular stress modalities: oxidative stress (H_2_O_2_), hypoxia, heat stress, and UV-induced DNA damage. All downstream analyses were performed independently within each stress modality to minimize biological heterogeneity and preserve stress-type-dependent transcriptional responses.

Differential expression analysis was subsequently performed to identify stress-responsive lncRNAs and protein-coding mRNAs, followed by GO enrichment analysis of DE protein-coding genes to characterize the biological processes associated with each stress condition. In parallel, WGCNA was used to identify treatment-associated co-expression modules. DE lncRNAs were then integrated with treatment-associated WGCNA modules to prioritize network-supported lncRNAs according to differential expression magnitude, module membership (|kME|), and module–treatment correlation (|r|).

The network-supported lncRNA were subsequently prioritized for candidates with recurrence across independent studies and cellular models. Finally, GO enrichment analysis of the retained candidate-containing WGCNA modules was performed to provide functional context for the prioritized candidates. Application of this analytical workflow identified a total of 377 lncRNAs candidates, comprising 329 oxidative stress-associated, 47 UV-induced DNA damage-associated, and one heat stress-associated lncRNAs, whereas no hypoxia-associated candidate lncRNA satisfied the integrated prioritization criteria. A study-level summary of the harmonized dataset, including GEO accession, NGS platform, cDNA library, stress modality, cell type, tissue of origin, treatment details, and control/treatment sample counts, is provided in Supplementary Table S1.

### 2.2. Dataset Heterogeneity, Cell-Type Composition, and Stress-Stratified Analysis

**Figure 2:**
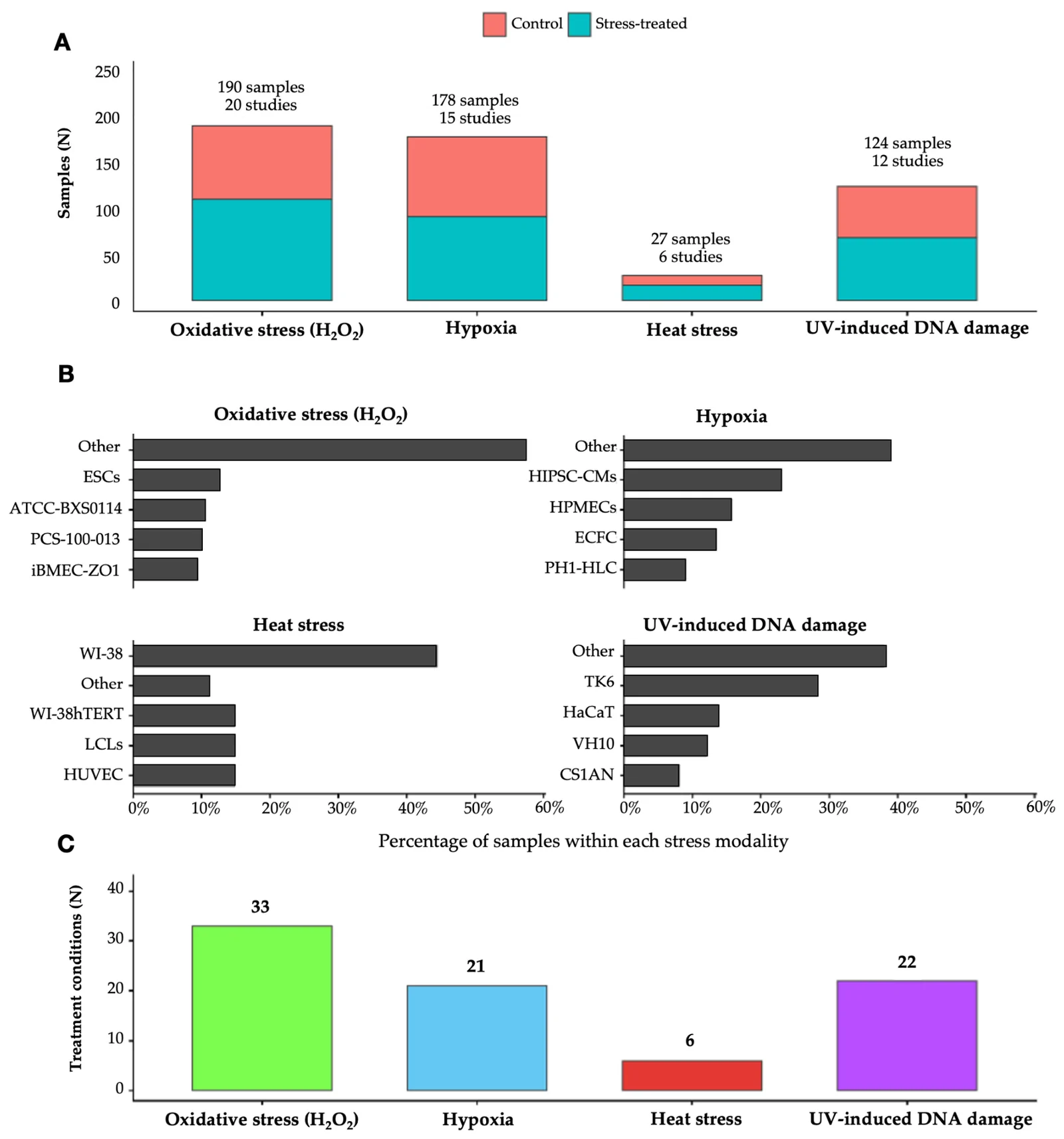
Dataset composition and experimental heterogeneity across stress modalities. (**A**) Distribution of control and stress-treated samples across the four stress modalities included in the analysis. The total sample size and the number of independent studies is indicated above each bar. (**B**) Major cell-type composition within each stress modality. For each stress type, the four most represented cell types are shown, while all remaining cell types are grouped as “Other”. Percentages were calculated relative to the total number of samples within each stress modality. (**C**) Diversity of treatment conditions within each stress modality, reflecting variation in the curated experimental parameters.

Dataset composition and experimental heterogeneity across the four stress modalities are summarized in Figure 2. The final harmonized dataset comprised 52 independent studies and 520 samples, distributed across H_2_O_2_-induced oxidative stress, hypoxia, heat stress, and UV-induced DNA damage. H_2_O_2_-induced stress represented the largest subset, with 20 studies and 190 samples, followed by hypoxia with 15 studies and 178 samples, UV-induced DNA damage with 12 studies and 124 samples, and heat stress with 6 studies and 27 samples. The complete harmonized dataset included 236 control samples and 284 stress-treated samples, supporting control–treatment comparisons within each stress modality (Supplementary Table S1).

To minimize data heterogeneity, our analysis was restricted to total RNA-seq experiments derived from normal, non-cancerous, wild-type human cell lines that had not undergone genetic manipulation, such as knock-out or knock-in. In addition, to minimize the impact of data heterogeneity, all downstream analyses were stratified according to stress modality, and edgeR-derived treatment effects were estimated exclusively from valid within-study control and treatment comparisons. Consequently, differences in baseline expression among unrelated studies or cell types were not interpreted as stress-induced effects. The primary objective of this study was to identify lncRNAs that are DE in response to a specific stressor in a recurrent and biologically meaningful manner across the analyzed datasets, rather than as universally conserved or cell-type-independent markers.

As discussed, the integrated datasets were heterogeneous in NGS platform, cellular context and treatment conditions, such as intensity and duration. The most represented cell types differed across stress modalities, including ESCs, ATCC-BXS0114, PCS-100-013, and iBMEC-ZO1 in oxidative stress/H_2_O_2_; HiPSC-CMs, HPMECs, ECFC, and PH1-HLC in hypoxia; WI-38, LCLs, HUVEC, and WI-38-hTERT in heat stress; and TK6, HaCaT, VH10, and CS1AN in UV-induced DNA damage (Figure 2B). Curated treatment annotations also revealed differences across experimental settings, with 33 distinct settings for oxidative stress/H_2_O_2_, 21 for hypoxia, 6 for heat stress, and 22 for UV-induced DNA damage (Figure 2C). Together, these descriptive statistics show that the integrated ASTRA/GEO dataset had to be experimentally heterogeneous to be broad. This, however, is a desirable structure to support our scientific hypothesis and enable the identification of stress-associated lncRNA-centered transcriptional programs that recur across heterogeneous cellular contexts.

### 2.3. Differential Expression Analysis Identifies Unique and Partially Shared lncRNAs Across Stress Modalities

**Figure 3:**
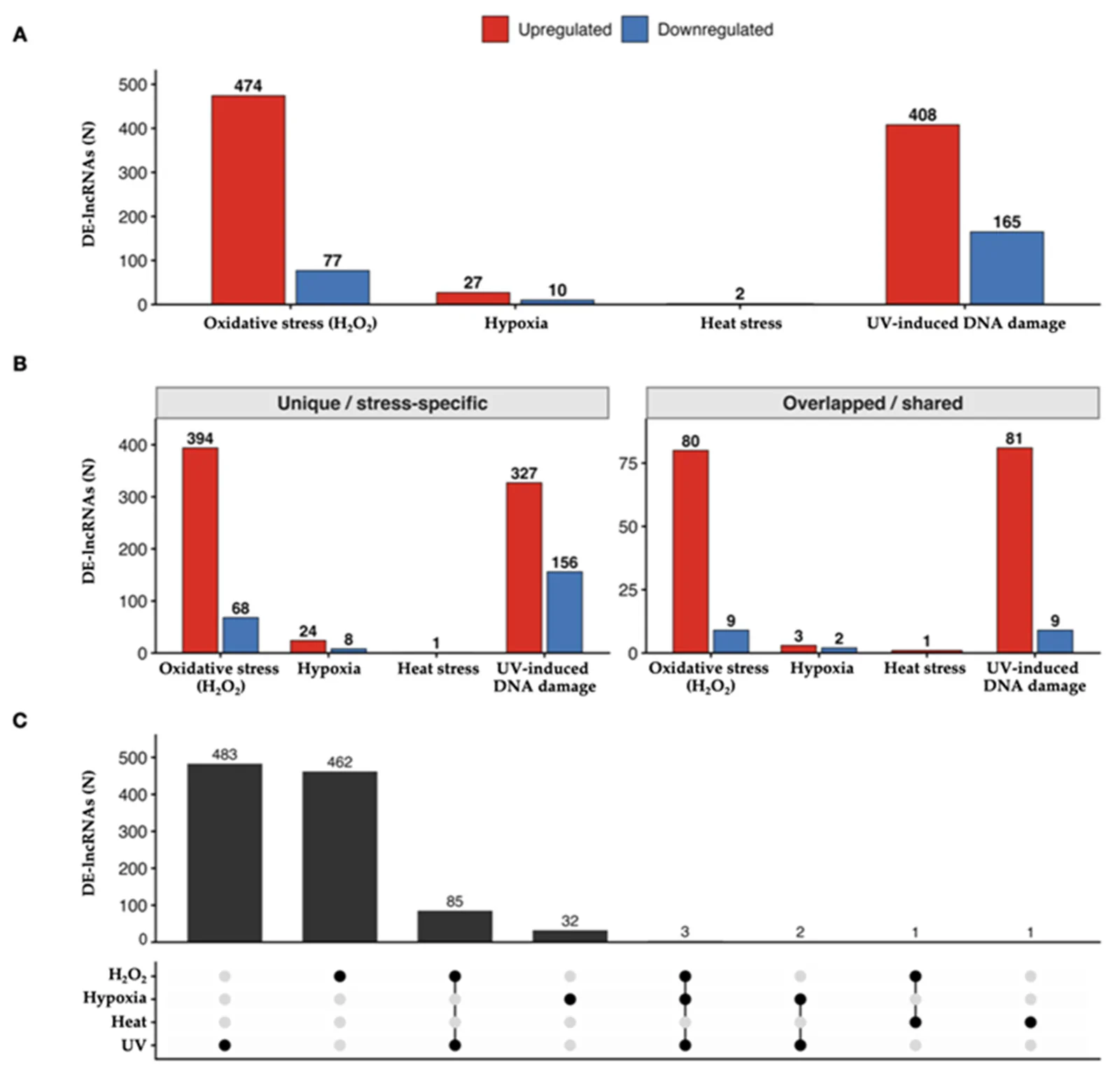
Stress type-dependent and shared DE-lncRNA profiles. (**A**) Number of DE lncRNAs identified in each stress modality, separated by direction of regulation. Upregulated lncRNAs are shown in red and downregulated lncRNAs in blue. DE lncRNAs were selected using an adjusted *p*-value threshold of <0.05 for H_2_O_2_-induced oxidative stress, hypoxia, and heat stress. For UV-induced DNA damage, where no transcripts passed adjusted *p*-value correction, lncRNAs were selected using a nominal *p*-value threshold of <0.05 and are therefore interpreted as an exploratory UV-associated signature. (**B**) Distribution of unique and overlapped/shared DE lncRNAs across stress modalities. Unique lncRNAs were defined as DE lncRNAs detected in only one stress modality. In contrast, overlapped/shared lncRNAs were defined as DE lncRNAs detected in two or more stress modalities. The direction of regulation is shown as in panel (**A**). (**C**) UpSet plot showing the intersection structure of DE lncRNAs across H_2_O_2_-induced oxidative stress, hypoxia, heat stress, and UV-induced DNA damage. Intersection bars indicate the number of lncRNAs shared by the corresponding stress modalities, while single-set intersections represent unique DE-lncRNAs.

Differential expression analysis was performed independently for each stress modality to identify stress-associated lncRNA transcriptional responses. For each stress modality, BatchQC generated a single aggregated differential-expression estimate for every transcript, consisting of an overall log_2_ fold-change together with its corresponding nominal and adjusted *p*-values, which served as the basis for all downstream differential-expression analyses. DE lncRNAs were selected using an adjusted *p*-value threshold of <0.05 for H_2_O_2_-induced oxidative stress, hypoxia, and heat stress. For UV-induced DNA damage, no lncRNA passed adjusted *p*-value correction; therefore, a nominal *p*-value < 0.05 was used to define an exploratory UV-associated signature (see Section 4). This stress-stratified strategy allowed us to distinguish lncRNAs selectively upregulated and potentially induced or suppressed in response to a particular molecular insult from lncRNAs that are DE in more than one stress type, as shown in Figure 3. The complete thresholded DE-lncRNA list, including differential expression statistics, direction of regulation, stress modality, and stress type-dependency or shared classification, is provided in Supplementary Table S2.

A total of 1069 DE lncRNAs were identified across the four stress modalities. H_2_O_2_-induced oxidative stress and UV-induced DNA damage displayed the largest lncRNA responses. H_2_O_2_ included 551 DE lncRNAs, of which 474 were upregulated, and 77 were downregulated. UV-induced DNA damage included 573 DE-lncRNAs, of which 408 were upregulated, and 165 were downregulated. In contrast, the hypoxia dataset comparison showed a more restricted lncRNA response, with 37 DE lncRNAs, including 27 upregulated and 10 downregulated transcripts. Heat stress showed the most limited response, with only 2 upregulated DE lncRNAs and no downregulated lncRNAs under the applied thresholding strategy. These results may indicate that redox imbalance and UV-induced genotoxic stress are associated with more extensive lncRNA transcriptional remodeling than hypoxia or heat stress in the analyzed datasets (Figure 3A).

We next used the same thresholded DE-lncRNA set to determine whether each transcript showed significant gene expression alterations to a unique or to more than one stress types. Unique lncRNAs were defined as DE lncRNAs detected in a single stress modality, whereas overlapped or shared lncRNAs were defined as DE lncRNAs detected in two or more stress modalities with a *p*-adjusted or nominal *p*-value threshold at *p* < 0.05. This analysis showed that the majority of DE lncRNAs were unique to one stress type. Overall, 978 lncRNAs were detected in only one stress modality, 88 were shared by two stress types, and only 3 were shared by three stress types. No lncRNA was shared across all four stress modalities (Supplementary Figure S1). The intersection structure of the thresholded DE lncRNA set was further examined using UpSet analysis (Figure 3C). At the individual stress level, H_2_O_2_ contained 462 stress type-dependent lncRNAs, UV-induced DNA damage contained 483, hypoxia contained 32, and heat stress contained 1 stress type-dependent lncRNA (Figure 3B). The largest intersection overlap was observed between H_2_O_2_ and UV-induced DNA damage, comprising 85 DE lncRNAs. This partial overlap may reflect common transcriptional features between H_2_O_2_- and UV-induced biological stress responses.

### 2.4. mRNA Enrichment Provides Pathway-Level Support for Stress Type-Dependent Transcriptional Programs

DE mRNAs within each stress modality were used as a pathway-level validation layer to assess whether our stress-stratified strategy captured biologically relevant stress-response transcriptional programs. DE mRNAs were analyzed by direction of regulation, and GO Biological Process enrichment analysis was performed independently on the upregulated and downregulated gene sets (Supplementary Table S4). The distribution of DE mRNAs varied markedly across stress modalities (Supplementary Figure S2 and Table S3). H_2_O_2_ showed the largest mRNA response, with 598 upregulated and 2680 downregulated genes. UV-induced DNA damage also showed a strong transcriptional response, with 347 upregulated and 714 downregulated mRNAs. However, hypoxia was characterized mainly by upregulation, with 265 upregulated and only 10 downregulated mRNAs. Heat stress showed the most restricted mRNA response, with 10 upregulated and no downregulated mRNAs under the applied thresholding strategy (adj *p* < 0.05).

**Figure 4:**
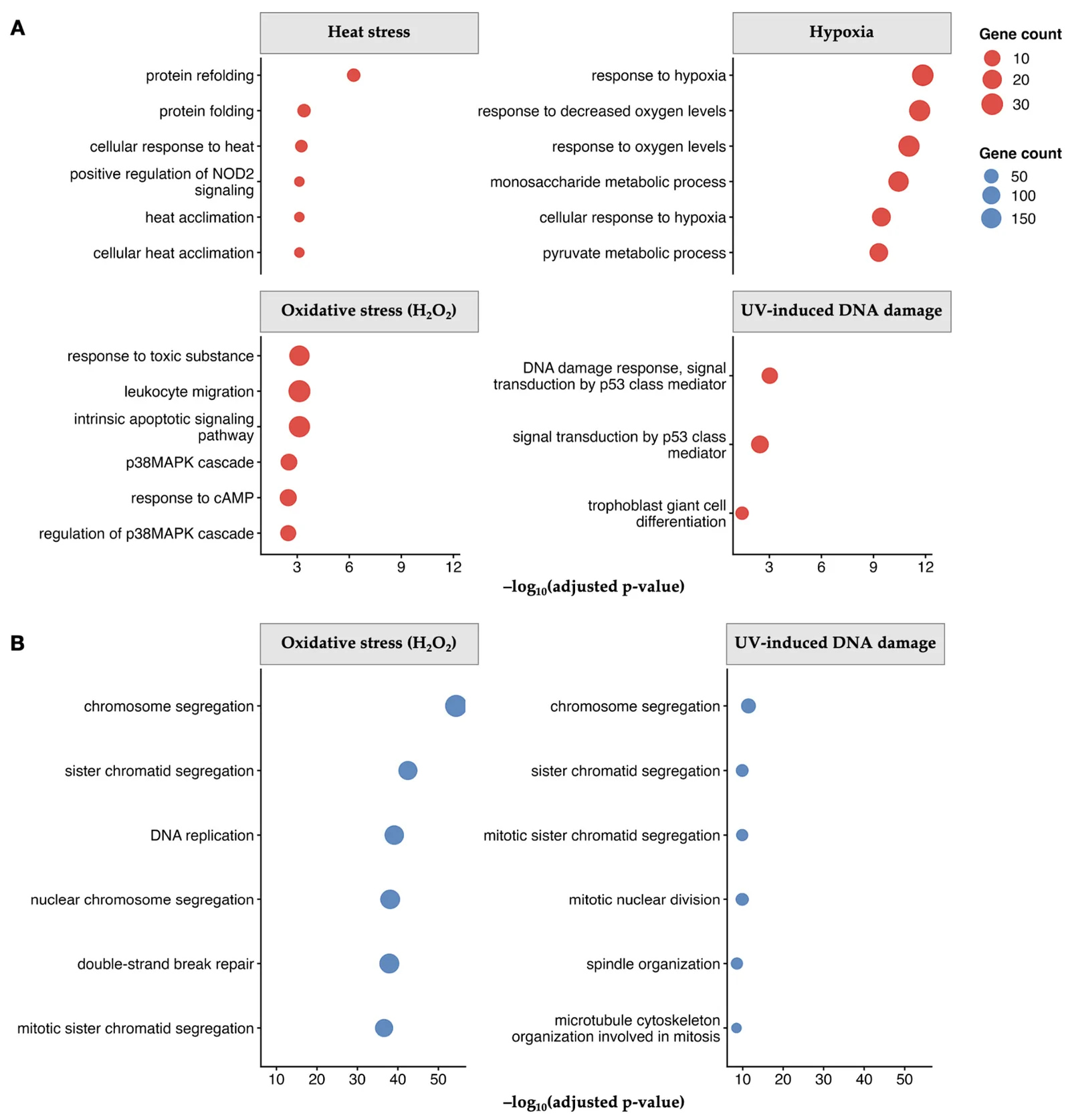
Functional annotation of stress-stratified differential expression using mRNAs. (**A**) The top 6 representative GO Biological Process terms enriched among upregulated mRNAs for each stress modality. Dot size represents the number of genes associated with each GO term, and the x-axis indicates −log_10_(adjusted *p*-value). (**B**) Top 6 representative GO Biological Process terms enriched among downregulated mRNAs in stress modalities with interpretable downregulated enrichment profiles. Dot size represents the number of genes associated with each GO term, and the x-axis indicates −log_10_(adjusted *p*-value). Full significant GO Biological Process enrichment results and the representative terms selected for visualization are provided in Supplementary Table S4.

GO Biological Process enrichment of DE mRNAs confirmed that each stress modality was associated with biologically coherent stress-response programs (Figure 4A,B). Among upregulated DE mRNAs, H_2_O_2_ was enriched for stress-response, inflammatory/migratory, and apoptosis-associated processes, including response to toxic substance (GO:0009636), intrinsic apoptotic signaling pathway (GO:0097193), leukocyte migration (GO:0050900), p38 MAPK cascade (GO:0038066), mononuclear cell migration (GO:0071674), and extrinsic apoptotic signaling pathway (GO:0097191). This profile is consistent with activation of cellular stress signaling and apoptosis-related responses under oxidative stress. Hypoxia-upregulated mRNAs were enriched for oxygen-sensing and metabolic adaptation processes, including response to hypoxia (GO:0001666), response to decreased oxygen levels (GO:0036293), response to oxygen levels (GO:0070482), cellular response to hypoxia (GO:0071456), monosaccharide metabolic process (GO:0005996), and pyruvate metabolic process (GO:0006090). This enrichment pattern supports the expected transcriptional adaptation to reduced oxygen availability and hypoxia-associated metabolic remodeling. Heat stress showed enrichment of proteostasis-related biological processes among upregulated mRNAs, including protein refolding (GO:0042026), protein folding (GO:0006457), cellular response to heat (GO:0034605), heat acclimation (GO:0010286), and response to heat (GO:0009408). These findings are consistent with activation of heat-shock and protein homeostasis mechanisms in response to thermal stress. UV-induced DNA damage was associated with enrichment of upregulated processes related to p53-mediated DNA damage signaling, including DNA damage response (GO:0030330), signal transduction by p53 class mediator (GO:0072331), and trophoblast giant cell differentiation (GO:0060707). This profile is consistent with activation of genotoxic stress-response signaling following UV-induced DNA damage.

Downregulated mRNA enrichment was observed mainly in H_2_O_2_ and UV-induced DNA damage (Figure 4B). In H_2_O_2_, downregulated genes were enriched for chromosome segregation (GO:0007059), sister chromatid segregation (GO:0000819), DNA replication (GO:0006260), nuclear chromosome segregation (GO:0098813), double-strand break repair (GO:0006302), and mitotic sister chromatid segregation (GO:0000070). Similarly, UV-induced DNA damage showed enrichment of downregulated processes related to chromosome segregation (GO:0007059), mitotic sister chromatid segregation (GO:0000070), sister chromatid segregation (GO:0000819), mitotic nuclear division (GO:0140014), spindle organization (GO:0007051), and microtubule cytoskeleton organization involved in mitosis (GO:1902850). These indicate the repression of proliferative, mitotic, and genome-maintenance programs, particularly under oxidative and genotoxic stress. In contrast, hypoxia showed no enriched downregulated GO Biological Process terms under the applied thresholding strategy, and heat stress showed no downregulated mRNAs.

The enrichment results confirm the biological specificity of the stress-stratified differential expression framework. Stress-associated transcriptional programs identified in each modality, independent of cellular context, aligned with the anticipated mRNA stress response to specific stimuli. These findings support the interpretation that DE lncRNAs in response to each stress type emerge within coherent, modality-specific cellular stress-response contexts.

### 2.5. WGCNA-Based Ranking of Network-Supported Stress-Associated DE lncRNA Candidates

To complement the differential expression analysis and determine whether DE stress-associated lncRNAs were embedded within coordinated co-expression networks, WGCNA was performed separately for each stress modality using the same complete harmonized dataset. Module eigengenes were correlated with treatment status, encoded as control = 0 and treated = 1. Because only one module passed FDR correction across the WGCNA analyses, treatment-associated modules were defined using a nominal module-trait *p*-value threshold of *p* < 0.05.

**Figure 5:**
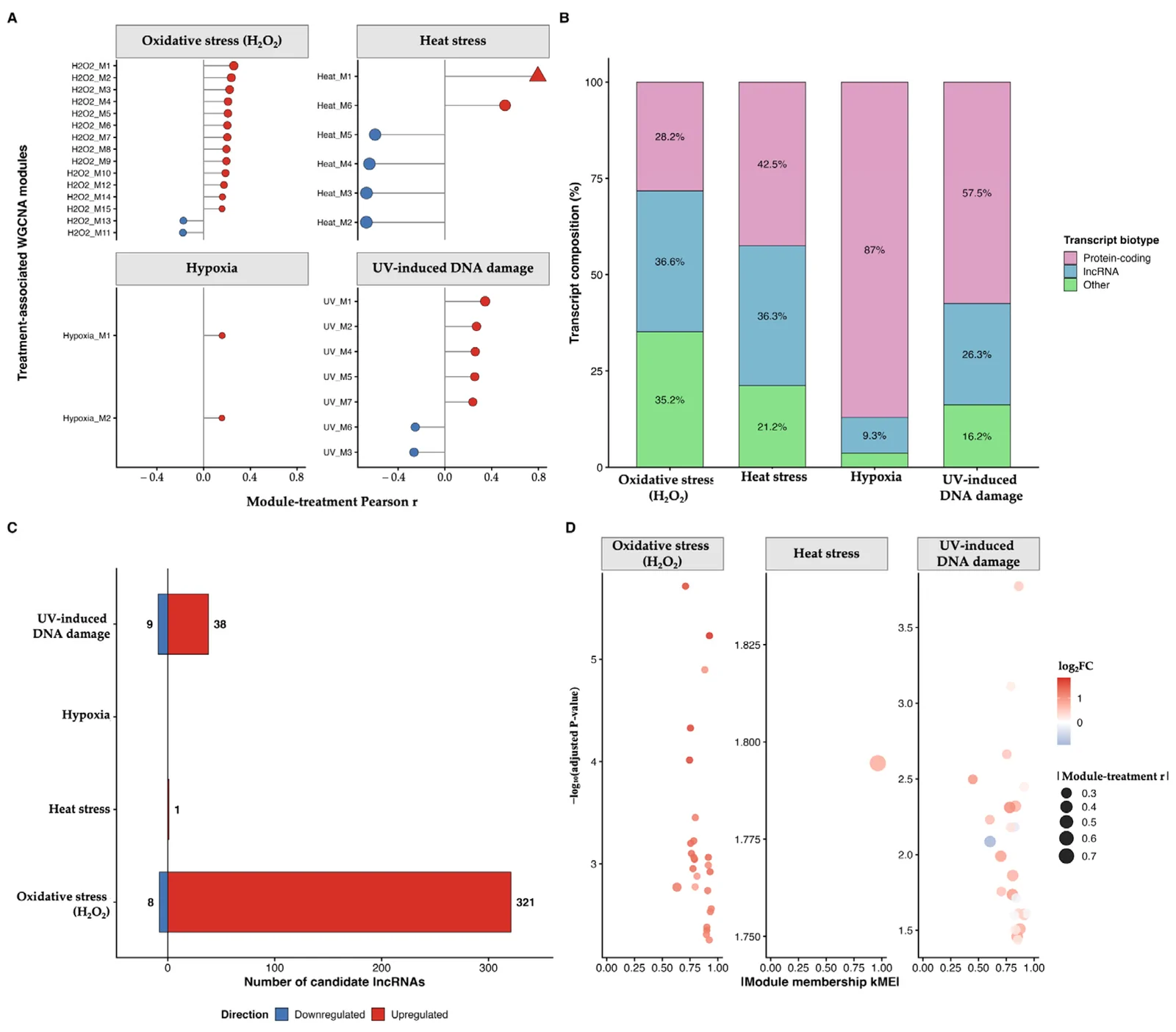
WGCNA-based ranking of network-supported stress-associated DE lncRNA candidates. (**A**) WGCNA module-treatment associations across stress modalities. Module eigengenes, defined as the first principal component of each module expression profile, were correlated with treatment status using Pearson correlation. Treatment status was encoded as control = 0 and treated = 1. The x-axis shows the module–treatment Pearson correlation coefficient; positive values indicate modules whose eigengene values are higher in treated samples, whereas negative values indicate modules whose eigengene values are higher in control samples. Only modules passing nominal module–trait *p* < 0.05 are shown. Point shape indicates statistical significance, distinguishing nominally significant modules from modules passing FDR correction. (**B**) Aggregated transcript biotype composition of nominally treatment-associated WGCNA modules. Bars show the percentage of protein-coding transcripts, lncRNAs, and other transcript biotypes across all treatment-associated modules within each stress modality. Numbers above each bar indicate the number of treatment-associated modules and the total number of transcripts included in the corresponding aggregated module set. (**C**) Number of lncRNA candidates identified by integrating differential expression analysis with WGCNA module membership. Candidate lncRNAs were defined as DE lncRNAs embedded within nominally treatment-associated WGCNA modules. Bars indicate the number of upregulated and downregulated candidate lncRNAs per stress modality. For H_2_O_2_-induced oxidative stress, heat stress, and hypoxia, DE lncRNAs were defined using an FDR-adjusted threshold of *p*adj < 0.05. For UV-induced DNA damage, where no lncRNAs passed FDR correction, an exploratory nominal threshold of *p* < 0.05 was used. Treatment-associated WGCNA modules were defined using module–trait *p* < 0.05. (**D**) Prioritization profile of top candidate lncRNAs. The x-axis shows absolute module membership, |kME|, indicating the strength of association between each lncRNA and its assigned WGCNA module. The y-axis shows DE significance, calculated as −log_10_(*p*adj) for H_2_O_2_, heat, and hypoxia candidates and as −log_10_(nominal *p*-value) for exploratory UV candidates. Bubble size represents the absolute module–treatment Pearson correlation, |r|, of the module containing each candidate lncRNA, while color indicates lncRNA log_2_FC. Higher |kME|, stronger DE significance, and larger |module–treatment r| indicate stronger network-supported candidate prioritization.

Across the four stress modalities, WGCNA identified distinct sets of treatment-associated co-expression modules (Figure 5A). Positive module–treatment correlations indicate modules with higher eigengene expression in treated samples, whereas negative correlations indicate lower eigengene expression relative to controls. Because both directions reflect treatment-associated transcriptional programs, positively and negatively correlated modules were retained for downstream analyses. Module–treatment correlation describes the association between module eigengenes and treatment status, whereas the direction of individual lncRNA regulation was inferred from edgeR-derived log_2_FC values.

Using the criterion, H_2_O_2_-induced oxidative stress showed the largest number of treatment-associated modules, with 15 modules. Heat stress was associated with 6 modules, including 2 positively and 4 negatively correlated modules: one of these also passed FDR correction. Hypoxia showed 2 treatment-associated modules, both positively correlated with treatment status. UV-induced DNA damage was associated with 7 modules, including 5 positively and 2 negatively correlated modules.

We next examined the transcript biotype composition of treatment-associated WGCNA modules to determine whether these co-expression structures contained substantial lncRNA representation. Aggregated across treatment-associated modules within each stress modality, lncRNAs represented a considerable fraction of the module transcript content (Figure 5B). In H_2_O_2_-associated modules, lncRNAs accounted for 36.6% of 11,261 transcripts across 15 modules, while mRNA transcripts represented 28.2% and other transcript biotypes 35.2%. Heat-associated modules contained 8593 transcripts across six modules, with 36.3% lncRNAs, 42.5% mRNA transcripts, and 21.2% other biotypes. Hypoxia-associated modules contained 162 transcripts across two modules and were dominated by mRNAs (87.0%), with lncRNAs representing 9.3% of the aggregated module content. UV-associated modules contained 2370 transcripts across seven modules, with 26.3% lncRNAs, 57.5% mRNAs, and 16.2% other biotypes.

To identify network-supported stress-associated lncRNAs, DE lncRNAs were intersected with nominally treatment-associated WGCNA modules with each stress modality. This integration identified 377 candidate lncRNAs across three out of four stress modalities (Figure 5C). H_2_O_2_ contributed the largest candidate set, with 329 candidate lncRNAs, including 321 upregulated and 8 downregulated candidates. These candidates were distributed across eight treatment-associated modules, with the largest fractions mapping to H_2_O_2__M4 (210 candidates), H_2_O_2__M9 (61 candidates), and H_2_O_2__M10 (39 candidates). UV-induced DNA damage contributed 47 candidate lncRNAs, including 38 upregulated and 9 downregulated candidates, distributed across six treatment-associated modules, primarily UV_M5 (23 candidates) and UV_M1 (14 candidates). Heat stress contributed one upregulated candidate lncRNA, *PARD6G-AS1*, assigned to Heat_M1. No hypoxia-associated candidate lncRNAs satisfied both differential-expression and network-support criteria under the applied framework analysis.

LncRNA candidates were subsequently ranked by integrating three complementary criteria: differential-expression magnitude, module membership (|kME|), and the absolute module–treatment correlation (|r|) of the corresponding WGCNA module (Figure 5D, Supplementary Table S5). Across the full candidate set, 80 lncRNAs were classified as high-kME candidates and 138 as moderate-kME candidates. H_2_O_2_ contained 63 high-kME and 119 moderate-kME candidates, UV contained 16 high-kME and 19 moderate-kME candidates, and the single Heat candidate was classified as high-kME. The top-ranked H_2_O_2_ candidates were primarily located in H_2_O_2__M4, whereas the top-ranked UV candidates were mainly distributed across UV_M5 and UV_M1. This multi-parameter ranking prioritized lncRNAs that combined differential expression with high intramodular connectivity and localization within treatment-responsive co-expression networks.

### 2.6. Cross-Study Recurrence Analysis Supports the Reproducibility of Prioritized Stress-Associated Transcripts

**Figure 6:**
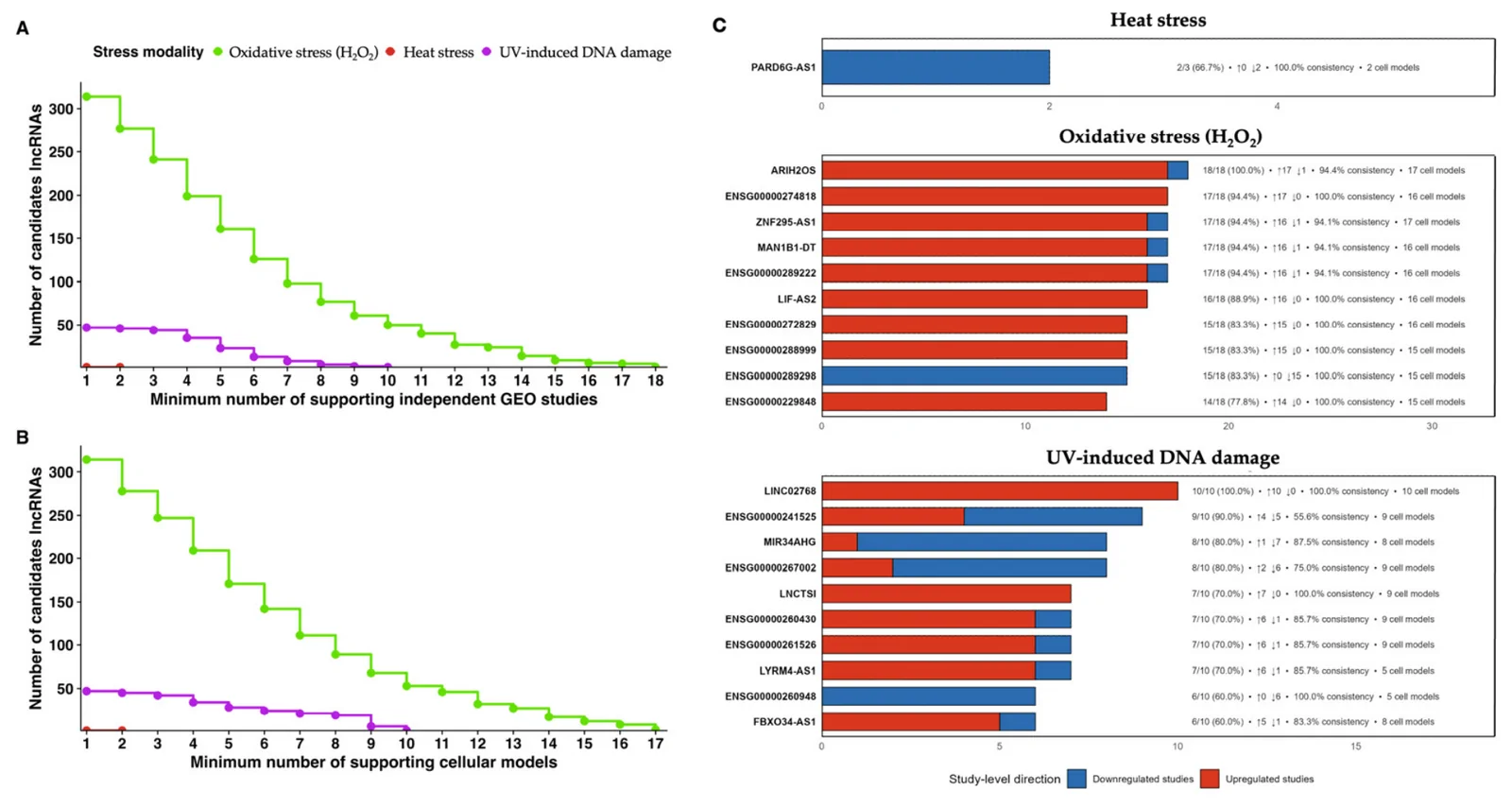
Cross-study recurrence analysis of prioritized candidate lncRNAs across independent GEO studies and cellular models. (**A**) Cumulative recurrence curves showing the number of prioritized candidate lncRNAs supported by increasing numbers of independent GEO studies for each stress modality. The x-axis indicates the minimum number of supporting independent studies, and the y-axis shows the number of candidate lncRNAs meeting or exceeding this threshold. (**B**) Cumulative recurrence curves showing the number of prioritized candidate lncRNAs supported by increasing numbers of distinct cellular models for each stress modality. The x-axis indicates the minimum number of supporting cellular models, and the y-axis shows the number of candidate lncRNAs meeting or exceeding this threshold. (**C**) Highest-ranked recurrent candidate lncRNAs for heat stress, H_2_O_2_-induced oxidative stress, and UV-induced DNA damage. Bar length represents the number of supporting independent GEO studies. Bar colours indicate the direction of differential expression across individual studies (red, upregulated; blue, downregulated). For each candidate, the recurrence percentage, numbers of upregulated and downregulated studies, directional consistency, and the number of supporting cellular models are shown. Candidate lncRNAs are ordered according to the number of supporting independent studies.

Following the identification of WGCNA-based ranked stress-associated DE lncRNAs, we next evaluated whether these candidates exhibited reproducible differential expression across the independent studies comprising each stress modality. lncRNA DE recurrence was evaluated based on the number of independent studies and distinct cellular models supporting differential expression, as well as the consistency of the observed direction of regulation across studies (Figure 6, Supplementary Table S6). Compared to lncRNAs, recurrent DE mRNAs were abundant across independent studies and cellular models. Oxidative stress (H_2_O_2_) exhibited the largest repertoire of recurrent DE mRNAs, followed by UV-induced DNA damage, whereas hypoxia and heat stress yielded comparatively fewer recurrent DE mRNAs. The highest-ranked DE mRNAs for each stress modality are summarized together with their recurrence across independent studies, directional consistency, and the number of supporting cellular models (Supplementary Figure S3).

LncRNAs showing a DE recurrence in at least two cell types and studies were prioritized. A total of 277 lncRNA candidates were retained for oxidative stress (H_2_O_2_), 46 for UV-induced DNA damage, and a single candidate (*PARD6G-AS1*) for heat stress (Figure 6A). The recurrence patterns differed markedly among stress modalities, with H_2_O_2_ exhibiting the broadest distribution of candidate lncRNAs across independent studies and cellular models, followed by UV-induced DNA damage. In contrast, the heat-stress candidate was identified in two of the three available studies (Figure 6A,B).

The highest prioritized candidates demonstrated substantial recurrence across independent datasets (Figure 6C). Within the H_2_O_2_ dataset, *ARIH2OS* emerged as the most recurrent candidate, being DE in all 18 independent studies and 17 cellular models, with a consistent upregulation of 94.4%. Additional highly recurrent candidates included *ZNF295-AS1*, *MAN1B1-DT*, *LIF-AS2*, and several unannotated lncRNAs, each supported by differential expression across 15–17 independent studies while maintaining directional consistency exceeding 94%. In the UV-induced DNA damage dataset, *LINC02768* was identified in all 10 available studies with 100% directional consistency, whereas *MIR34AHG*, *LNCTS1*, and *LYRM4-AS1* were also recurrently detected across multiple independent studies and cellular models. Heat stress retained a single candidate (*PARD6G-AS1*), which was consistently upregulated in two of the three available studies, reflecting both the limited dataset size and the more restrictive candidate-selection outcome for this stress modality. Together, these findings indicate that the highest prioritized network-supported lncRNA candidates were not driven by single datasets but instead represented recurrent stress-associated transcriptional events reproducibly observed across independent experimental studies.

### 2.7. Retained WGCNA Modules Reveal the Functional Context of the Prioritized lncRNA Candidates

**Figure 7:**
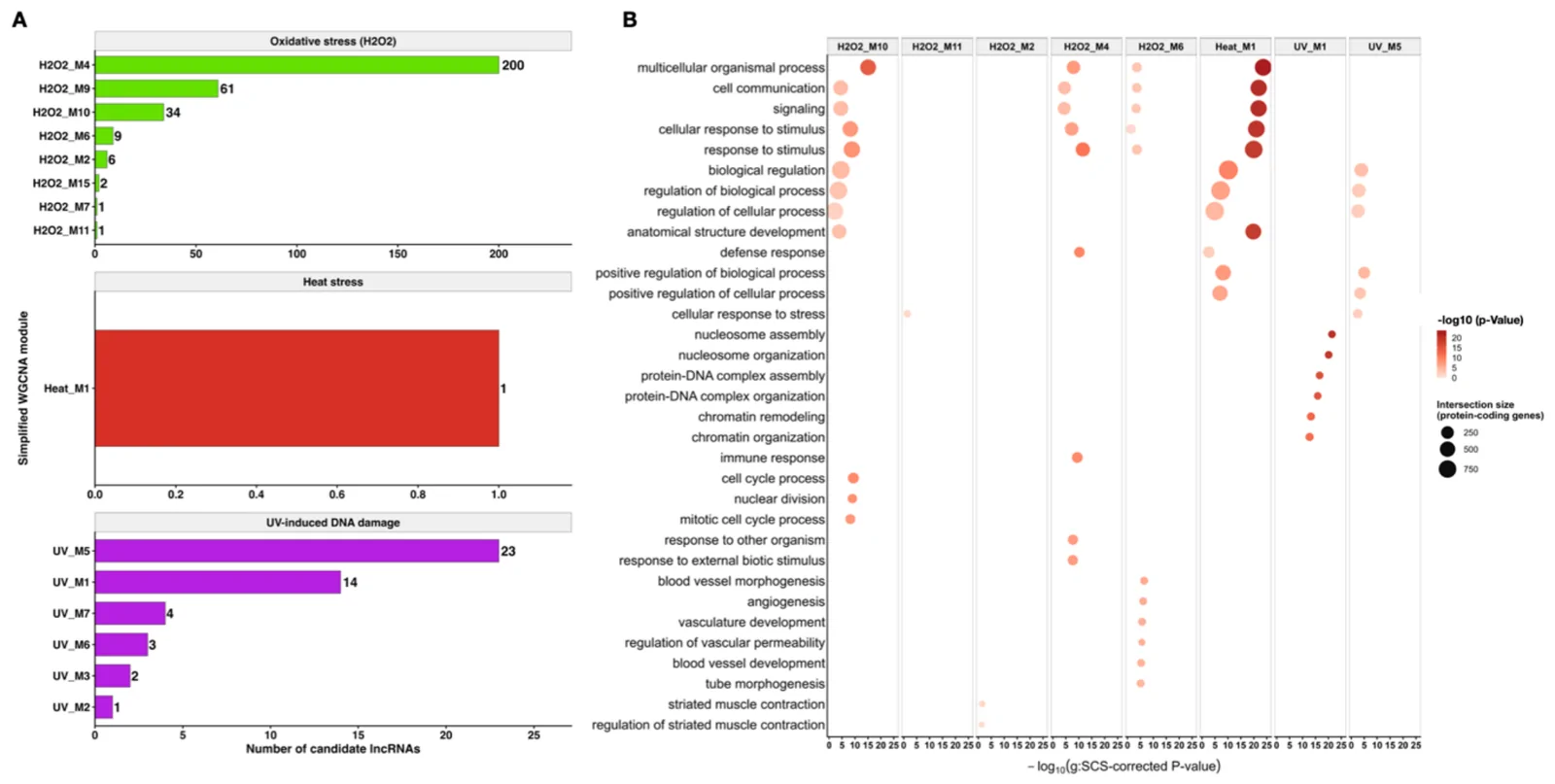
Retained candidate-containing WGCNA modules reveal the functional context of prioritized stress-associated lncRNAs. (**A**) Distribution of prioritized candidate lncRNAs across the retained treatment-associated WGCNA modules following integration of differential expression and co-expression analyses. Only retained modules containing prioritized candidate lncRNAs were subjected to downstream functional annotation. Bars indicate the number of candidate lncRNAs assigned to each retained module within oxidative stress (H_2_O_2_), heat stress, and UV-induced DNA damage. No hypoxia-associated candidate lncRNAs satisfied the integrated prioritization criteria. (**B**) Gene Ontology (GO) Biological Process enrichment analysis of protein-coding genes contained within the retained candidate-containing WGCNA modules. Bubble size represents the number of protein-coding genes associated with each enriched GO term (intersection size), whereas color intensity indicates enrichment significance as −log_10_(SCS-corrected *p* value). The enrichment profiles demonstrate that prioritized candidate lncRNAs are embedded within biologically coherent and stress type-depedent co-expression modules associated with distinct cellular processes, including cell-cycle regulation, immune and inflammatory responses, vascular development, heat-responsive signaling, and chromatin organization following UV-induced DNA damage.

To provide functional context for the prioritized candidate lncRNAs, we first determined their distribution across the treatment-associated WGCNA modules identified for each stress modality. Rather than being uniformly distributed, candidate lncRNAs clustered within a limited number of modules (Figure 7A). Consequently, only these candidate-containing modules were retained for downstream functional annotation.

Among the oxidative stress networks, candidate lncRNAs were predominantly localized within module H_2_O_2__M4 (n = 200), followed by H_2_O_2__M9 (n = 61) and H_2_O_2__M10 (n = 34), whereas only a small number of candidates were identified in modules H_2_O_2__M6 (n = 9), H_2_O_2__M2 (n = 6), H_2_O_2__M15 (n = 2), H_2_O_2__M7 (n = 1) and H_2_O_2__M11 (n = 1). Heat stress exhibited a single retained candidate-containing module (Heat_M1), whereas UV-induced DNA damage was characterized mainly by modules UV_M5 (n = 23) and UV_M1 (n = 14), with only a few candidates detected in UV_M7, UV_M6, UV_M3 and UV_M2 (Figure 7A).

To infer the biological programs associated with these prioritized lncRNAs, GO Biological Process enrichment analysis was subsequently performed using the protein-coding genes contained within the retained candidate-containing modules (Figure 7B, Supplementary Table S8). The enriched biological processes demonstrated that prioritized lncRNAs were embedded within biologically coherent and stress type-dependent transcriptional networks rather than randomly distributed across the co-expression landscape.

Within the oxidative stress network, the retained modules collectively represented multiple complementary biological programs. Module H_2_O_2__M10 was enriched for processes related to cell-cycle progression (GO:0022402; cell cycle process), mitotic progression (GO:0000280; nuclear division, GO:1903047; mitotic cell cycle process), and broader cellular responses to external stimuli (GO:0051716, cellular response to stimulus; GO:0042221, response to chemical), collectively indicating coordinated regulation of cell-cycle progression and proliferative responses under oxidative stress. In contrast, H_2_O_2__M4, which contained the largest number of prioritized candidate lncRNAs, was primarily associated with inflammatory and innate immune programs, including response to stress (GO:0006950), immune response (GO:0006955), inflammatory response (GO:0006954), cytokine-mediated signaling (GO:0019221), and responses to bacterial-derived molecules such as lipopolysaccharide (GO:0032496) and molecules of bacterial origin (GO:0002237). Module H_2_O_2__M6 was enriched for biological processes related to vascular development and angiogenesis, including vasculature development (GO:0001944), blood vessel development (GO:0001568), blood vessel morphogenesis (GO:0048514) and angiogenesis (GO:0001525), and regulation of vascular permeability and angiogenesis (GO:0043114, GO:0045765), while the smaller modules displayed more restricted functional signatures, including cellular response to stress (H_2_O_2__M11; GO:0033554) and striated muscle contraction (H_2_O_2__M2; GO:0006941).

The single retained heat-stress module (Heat_M1) showed enrichment for broad biological processes associated with heat response, including protein folding and refolding (GO:0006457, GO:0042026) and responses to heat, temperature stimulus, and unfolded protein (GO:00009408, GO:0009266, GO:0006986). Beyond the canonical heat-shock response, Heat_M1 was also enriched for cell communication and signal transduction (GO:0007154, GO:0007165), together with developmental programs including cell development (GO:0048468), cell differentiation (GO:0030154), anatomical structure morphogenesis (GO:0009653), and response to growth factors (GO:007155), were also detected, indicating that prioritized heat-responsive lncRNAs were embedded within coordinated adaptive signaling networks.

In the UV-induced DNA damage condition, the two major retained modules exhibited distinct biological functions. UV_M1 was predominantly enriched for chromatin organization and nucleosome dynamics, including nucleosome assembly and organization (GO:0006334, GO:0034728), protein-DNA complex organization (GO:0071824), chromatin remodeling (GO:0006338), and epigenetic regulation of gene expression (GO:0040029), reflecting extensive chromatin reorganization following genotoxic stress. Conversely, UV_M5 was enriched for higher-order biological regulation (GO:0065007), positive regulation of biological processes (GO:0048518), positive regulation of cellular processes (GO:0048522), and cellular response to stress (GO:0033554).

Collectively, these findings demonstrate that network-supported DE lncRNAs in at least 2 different cell types are concentrated within a restricted subset of treatment-associated co-expression modules representing biologically coherent and stress type-dependant functional programs. This supports that our network-ranking and recurrency of DE framework analysis captures stress-associated mRNAs and lncRNAs that are regulated more by the molecular stress type and less by cellular context, representing stress type-dependent candidate markers.

## 3. Discussion

In this study, we carried out an integrative RNA-seq analysis to examine the extent to which lncRNAs are regulated in a stress-dependent or cell-context independent manner, using mRNAs as a transcript biotype reference group. By combining datasets from ASTRA and GEO, harmonizing the associated metadata, and analyzing each stress modality separately, we developed a stress-stratified framework for identifying and prioritizing stress-associated lncRNA-centered transcriptional responses. The analysis focused on four major forms of primary molecular stresses: H_2_O_2_-induced oxidative stress, hypoxia, heat stress, and UV-induced DNA damage. Across all four treatments, lncRNAs represented a substantial component of the stress-responsive transcriptome, reinforcing their role as state-dependent regulators and stress-induced molecular indicators of cellular adaptation.

One of the main findings of this study is that lncRNA responses to molecular stress are largely stress-type-dependent. Among the 1069 DE lncRNAs identified across the four stress modalities, most appeared in only one condition. Only a small proportion was shared between two or three stress types, and no DE lncRNA was common to all four. These results suggest that lncRNAs’ responses to stress do not collapse into a broad, universal pan-stress signature under the conditions analyzed here. Instead, they seem to be mostly activated under the specific molecular challenges imposed by each stressor. H_2_O_2_-induced oxidative stress and UV-induced DNA damage showed the broadest lncRNA upregulation, whereas hypoxia and heat stress produced, in general, limited signatures. This pattern may reflect the fact that redox imbalance and genotoxic stress activate a wide range of pathways, including DNA damage signaling, apoptosis, cell-cycle checkpoints, inflammatory responses, and genome maintenance [18,22,34,35,36].

An important feature of the stress type-dependent transcriptional programs identified here is that they emerged across datasets derived from different studies and cellular contexts. Therefore, stress type-dependent lncRNAs should not be interpreted simply as transcripts detected within a single uniform experimental system; rather, their recurrence within a given stress modality suggests that at least a subset of lncRNA-mediated responses may represent stress-modality-conserved transcriptional features that are robust enough to be detected despite differences in cell type, laboratory of origin, and treatment design. The predominance of upregulated stress type-dependent lncRNAs further suggests that molecular stressors may actively induce non-coding transcriptional programs across cellular contexts. These stress-induced lncRNAs may therefore provide sensitive readouts of how human cells respond to distinct molecular insults and of the adaptive mechanisms activated in each stress state. Oxidative stress-associated lncRNAs may be linked to redox-sensitive signaling, inflammatory activation, apoptosis, or detoxification pathways [37,38]; hypoxia-associated lncRNAs may reflect oxygen sensing and metabolic adaptation [20,39]; heat-associated lncRNAs may relate to proteostasis and heat-shock responses [21,32]; and UV-associated lncRNAs may be connected to DNA damage signaling and checkpoint control [34,40]. Although the UV-induced DNA damage signature was retained using a nominal *p*-value threshold and should therefore be considered exploratory, its partial overlap with the H_2_O_2_-induced oxidative stress signature suggests that oxidative and genotoxic stress share selected non-coding transcriptional features [41,42], while also showing repression of proliferative mRNA programs [40,43].

Another strength of this study is the use of ASTRA as a structured framework for integrating stress-response transcriptomic data. By organizing datasets according to stress factor, treatment condition, cell type, tissue origin, and transcript biotype, ASTRA enabled the systematic selection and harmonization of public RNA-seq studies relevant to human cellular stress. This was especially important given the marked heterogeneity of the available datasets, including differences in cell-line background, exposure duration, stress intensity, treatment protocol, and experimental design [33]. Rather than treating molecular stress as a single uniform condition, the ASTRA-based metadata structure allowed each stress modality to be analyzed separately and made it possible to distinguish stress type-dependent from partially shared stress-associated lncRNA-centered transcriptional programs. In this setting, ASTRA did not represent the main biological finding of the study, but rather the analytical scaffold that made comparative stress biology possible and supported the prioritization of candidate lncRNAs linked to stress-adaptive cellular states.

Integrating differential expression with WGCNA added a network-level layer to candidate prioritization. Whereas differential expression identifies stress-responsive lncRNAs, co-expression analysis helps determine whether these transcripts are embedded within coordinated stress-associated transcriptional programs. Thus, lncRNAs retained after the DE–WGCNA intersection are supported not only by reproducible gene-level deregulation but also by module-level organization, making them stronger candidates for downstream functional investigation. Functional annotation of the retained treatment-associated modules further demonstrated that prioritized candidate lncRNAs were embedded within biologically coherent stress-related processes, thereby supporting the biological relevance of the network-based prioritization strategy. The subsequent cross-study robustness analysis further strengthened this prioritization by identifying recurrent candidates consistently detected across independent studies and cellular models, thereby increasing confidence in their biological relevance. The absence of retained hypoxia-associated candidates under the applied thresholds may reflect a more restricted lncRNA response, stronger dependence on cellular context, or reduced capture by the current network criteria, rather than the absence of lncRNA involvement in hypoxic adaptation.

This network-supported prioritization strategy aligns with recent studies showing that stress-related lncRNAs can be biologically interpreted through their association with protein-coding gene programs. For instance, Shi et al. identified oxidative stress-related lncRNAs in glioma by combining oxidative stress-associated genes, lncRNA-mRNA correlation analysis, functional enrichment, prognostic modeling, immune infiltration analysis, and RT-qPCR validation [44]. Although that work focused on glioma prognosis rather than multi-stress cellular responses, it supports the broader idea that biologically relevant lncRNAs can be prioritized based on their connections to coordinated stress-associated gene networks rather than on differential expression alone. Similarly, our framework integrated differential expression and treatment-associated co-expression modules for candidate prioritization, followed by functional annotation and cross-study robustness assessment to further characterize the retained candidates within biologically meaningful stress-response programs.

An additional direction for future work will be the dedicated analysis of shared lncRNA-associated transcriptional modules. Although the present study classified DE lncRNAs as stress type-dependent or shared across two or three stress modalities, the downstream network analysis focused primarily on stress-stratified candidate prioritization within each modality. Future studies should examine whether shared DE lncRNAs are embedded within conserved or convergent co-expression modules across stress types, and whether these modules represent common adaptive programs, such as repression of proliferation, genome maintenance, proteostasis remodeling, or inflammatory stress signaling. In this context, hub-like candidates such as *PARD6G-AS1*, which was prioritized in heat stress and was also upregulated in H_2_O_2_- and UV-associated stress contexts, may be particularly promising targets for mechanistic studies investigating whether shared lncRNAs acquire stress type-dependent network roles and contribute to the regulation of heat-stress-associated mRNA programs compared with other stress modalities.

Several limitations should be considered when interpreting these findings. First, the integrated dataset was heterogeneous with respect to the GEO study, cell type, treatment duration, stress intensity, and experimental design. Although this heterogeneity reflects the diversity of publicly available datasets and makes it possible to identify recurring stress-associated programs across cellular contexts, it also introduces batch and context effects that cannot be fully removed. To minimize this issue, analyses were carried out separately for each stress modality, and batch effects were assessed and corrected where appropriate. Second, the UV-induced DNA damage dataset did not yield FDR-significant DE lncRNAs and was therefore analyzed using a nominal *p*-value threshold. Consequently, UV-associated lncRNAs should be regarded as exploratory and hypothesis-generating rather than high-confidence DE candidates. Third, the functional interpretation of lncRNAs was inferred indirectly through mRNA, GO enrichment, module membership, and co-expression patterns. While these analyses provide pathway-level biological support, they do not replace experimental functional validation.

Despite these limitations, the present analysis provides a modular framework for prioritizing candidate regulatory lncRNAs involved in physiological human cellular stress responses, with potential extension to stress-related disease contexts such as cancer. By integrating differential expression, stress type-dependent overlap and WGCNA module association, we classified lncRNAs into stress type-dependent, partially shared, and network-supported candidates. Importantly, prioritization should not be interpreted as a rigid exclusion system, since biologically relevant lncRNAs may also be present among moderate-priority candidates. In support of this, *KDM7A-DT*, a stress-induced promoter-associated antisense lncRNA previously characterized by our group, was experimentally validated in breast cancer models through oxidative-stress induction, knockdown-based functional analysis, and mechanistic links to DNA damage response/repair, apoptosis regulation, G2/M checkpoint control, and cancer-associated transcriptional programs [29]. Although *KDM7A-DT* does not fall within the highest-confidence candidate class in the present framework, it has an increased recurrence (78%) and consistency of regulation (93%), supporting the relevance and reproducibility of moderate-priority lncRNAs and suggesting that this strategy may capture stress type-dependent candidates beyond normal cellular stress models.

## 4. Materials and Methods

### 4.1. Data Retrieval from ASTRA and GEO and Metadata Harmonization

RNA-seq datasets available through ASTRA, together with additional recently published datasets identified using Public Omics Explorer (POE) https://nplab.gr/poe (accessed on 20 June 2026) [45], were retrieved and systematically curated for downstream analysis. Sequencing reads were trimmed and quality-filtered using fastp (v1.3.6) [46], aligned to the Homo sapiens GRCh38.dna primary assembly reference genome using STAR (v2.7.11) [47], and alignment quality was summarized using MultiQC (v1.34) [48]. In total, 795 samples were processed at the alignment stage, of which 519 samples passed downstream inclusion criteria and were retained for the integrated analyses (Supplementary Table S1). Across the aligned samples, the median unique mapping rate was approximately 87.5% (IQR: 85–90%), indicating overall high alignment quality. Reads mapping to multiple genomic loci represented a minor fraction of the data, with a median of approximately 5.5%, whereas reads mapping to excessive numbers of loci or falling into other unmapped categories accounted for less than 0.5% of total reads.

Given the heterogeneous multi-study design, batch effects were evaluated and addressed before downstream analyses. The included datasets differed substantially in cell line, laboratory of origin, treatment implementation, exposure duration, and stress modality. In many cases, GEO study identity was strongly confounded with cell type and treatment setting, making cross-study batch effects an expected feature of the integrated dataset. To reduce this heterogeneity, all downstream analyses were performed separately within each stress modality.

BatchQC (v2.8.0) [49] in R (v4.5.3) was used to assess the relationship between metadata variables and study identity, using the GEO accession as the batch variable and condition, defined as control versus stress-treated, as the biological covariate. Raw count matrices were used as input for diagnostic assessment and normalization. For exploratory visualization and co-expression analyses, raw counts were normalized to log-CPM values and batch-corrected using ComBat with a parametric empirical Bayes framework, as implemented in the BatchQC Shiny app environment. Batch correction was performed with GEO study identity as the batch variable and condition as the biological variable. These batch-corrected log-CPM matrices were subsequently used for PCA-based assessment and WGCNA analyses.

### 4.2. Differential Expression Analysis with edgeR

Differential expression analysis was performed independently for each stress modality using edgeR (v4) [50]. Raw count matrices for each stress modality were analyzed in the BatchQC Shiny environment, using the GEO study accession as the batch variable and the treatment condition as the biological variable of interest. Raw count data were normalized using the Trimmed Mean of M-values (TMM) method implemented in edgeR. Differential expression was assessed using generalized linear models (GLM) with quasi-likelihood F-tests implemented in edgeR, as provided within the BatchQC analysis workflow. Benjamini–Hochberg correction was applied to control the false discovery rate, and adjusted *p*-values were used for statistical filtering.

For each stress modality, BatchQC generated an aggregated differential-expression result after batch adjustment, yielding a single log_2_ fold-change estimate together with the corresponding nominal *p*-value and Benjamini–Hochberg-adjusted *p*-value for each transcript. These aggregated differential-expression estimates were used throughout the downstream candidate-identification and prioritization pipeline. In parallel, the individual study-level edgeR outputs were retained and subsequently used exclusively for the independent recurrence and robustness analyses.

For oxidative stress, heat stress, and hypoxia, transcripts with adjusted *p*-value < 0.05 and log_2_FC (log_2_FoldChange) > 0 were considered upregulated, whereas transcripts with adjusted *p*-value < 0.05 and log_2_FC < 0 were considered downregulated. For UV-induced DNA damage, no lncRNAs passed the adjusted (Benjamini–Hochberg) threshold; therefore, UV-associated lncRNAs were retained using an exploratory nominal *p*-value threshold of *p* < 0.05. This UV signature was interpreted as exploratory and was distinguished from the BH-supported signatures obtained for the other stress modalities.

DE transcripts were stratified according to transcript biotype using the ASTRA annotation file generated from Ensembl BioMart through the biomaRt Bioconductor package (v3.22) [51]. DE lncRNAs and protein-coding transcripts were subsequently used in the downstream analyses described below. Upregulated and downregulated protein-coding gene sets were analyzed separately for each stress modality (Supplementary Tables S2 and S3).

### 4.3. Stress Type-Dependent Weighted Gene Co-Expression Network Analysis

WGCNA was performed separately for each stress modality using the WGCNA package in R [52]. For each stress type dataset, normalized batch-corrected expression matrices were used to construct signed co-expression networks. Soft-thresholding powers were selected based on scale-free topology criteria while preserving adequate mean network connectivity. The selected soft-thresholding powers were 6 for H_2_O_2_, 12 for heat stress, 6 for hypoxia, and 10 for UV-induced DNA damage. Heat stress did not fully reach the scale-free topology threshold; therefore, the selected power represented the best compromise between scale-free topology fit and mean connectivity for this dataset.

Signed adjacency matrices were transformed into signed topological overlap matrices (TOMs), and transcript modules were detected using hierarchical clustering followed by dynamic tree cutting, with a minimum module size of 30 transcripts. The whole transcriptome was used for network construction, with block sizes of 10,000 transcripts. To avoid over-splitting of highly similar modules, modules with eigengene correlation ≥ 0.75, corresponding to a merge Cut Height of 0.25, were merged.

Module eigengenes were correlated with treatment status using Pearson correlation. Treatment status was encoded as a binary trait based on the curated metadata, with control samples coded as 0 and stress-treated samples coded as 1. For each module-trait correlation, a corresponding *p*-value was calculated. Modules with nominal *p* < 0.05 were considered treatment-associated and were retained for downstream analyses. Both positively and negatively correlated modules were considered treatment-associated, as they represent co-expression programs whose eigengene values vary with treatment status.

For treatment-associated modules, transcript biotype composition was assessed, and protein-coding transcripts within these modules were used for Gene Ontology [53] Biological Process enrichment to characterize the biological context of stress-associated co-expression programs.

### 4.4. Functional Enrichment of DE Protein-Coding Transcripts

DE transcripts obtained from edgeR were filtered according to stress type-dependent statistical thresholds. For H_2_O_2_-induced oxidative stress, heat stress, and hypoxia, transcripts were considered DE using an adjusted *p*-value threshold of *p*adj < 0.05. For UV-induced DNA damage, where no lncRNAs passed adjusted *p*-value correction, an exploratory nominal threshold of *p* < 0.05 was used. DE transcripts were stratified by transcript biotype and direction of regulation.

For GO functional validation of the stress-stratified differential expression results, mRNA transcripts were analyzed separately from lncRNAs. DE mRNAs were divided into upregulated and downregulated subsets for each stress modality, and Gene Ontology Biological Process enrichment analysis was performed separately for each subset. The heat stress dataset did not contain downregulated protein-coding transcripts passing the applied adjusted *p*-value threshold and was therefore not included in the downregulated enrichment analysis. Enrichment significance was assessed using Benjamini–Hochberg correction, and GO Biological Process terms with adjusted *p*-value < 0.05 were considered significant. Full enrichment results are provided in Supplementary Table S4.

### 4.5. Integration of Differential Expression and Co-Expression Network Analysis for Candidate lncRNA Prioritization

To prioritize candidate stress-associated lncRNAs, differential expression analysis and WGCNA results were integrated separately for each stress modality. Differential expression analysis was used to identify lncRNAs whose expression changed upon stress exposure, whereas WGCNA was used to identify co-expression modules significantly associated with treatment status. lncRNAs were considered candidate stress-associated molecules when they were both DE and assigned to WGCNA modules showing nominal association with treatment status, defined as module-trait *p* < 0.05.

For H_2_O_2_-induced oxidative stress, hypoxia, and heat stress, DE lncRNAs were defined using an adjusted *p*-value threshold (*p*adj < 0.05). For UV-induced DNA damage, where no lncRNAs passed adjusted *p*-value correction, an exploratory nominal threshold of *p* < 0.05 was used. Candidate lncRNAs were further annotated according to stress modality, differential expression direction, WGCNA module assignment, module membership, module–treatment correlation, module composition, and prioritization class. The full DE–WGCNA candidate lncRNA table is provided in Supplementary Table S5.

### 4.6. Cross-Study Robustness Analysis of Prioritized Candidate lncRNAs and Differentially Expressed Protein-Coding Genes

To evaluate the reproducibility of the prioritized candidate lncRNAs across independent studies, a study-level robustness analysis was performed using the individual study-level differential expression results generated by the BatchQC workflow for each GEO dataset within each stress modality. Differential expression analyses were performed independently for each study using the same statistical criteria applied throughout the study (adjusted *p* < 0.05 for oxidative stress, hypoxia and heat stress; nominal *p* < 0.05 for UV-induced DNA damage).

For each prioritized candidate lncRNA, the number of independent supporting studies, the number of supporting cellular models, the predominant direction of differential expression (upregulated or downregulated), and the directional consistency across studies were calculated. Study recurrence was additionally expressed as the percentage of supporting studies relative to the total number of available studies for the corresponding stress modality (Supplementary Table S6).

The same robustness analysis was independently applied to all DE protein-coding genes identified within each stress modality, irrespective of WGCNA module assignment, using identical recurrence and directional consistency metrics. The robustness analysis was implemented in R (version 4.4.0) using custom scripts developed for this study (Supplementary Table S7).

### 4.7. Functional Annotation of Retained Candidate-Containing WGCNA Modules

To functionally characterize the retained treatment-associated WGCNA modules containing prioritized candidate lncRNAs, GO Biological Process (GO:BP) enrichment analysis was performed using the protein-coding genes assigned to each retained module.

Functional enrichment analyses were performed independently for each retained module in R (version 4.4.0) using the gprofiler2 package (version 0.2.4), which interfaces with the gProfiler functional enrichment framework. Protein-coding gene lists corresponding to each retained module were queried against the GO:BP database. Statistical significance was assessed using the built-in gSCS multiple-testing correction implemented in gProfiler, and significantly enriched GO:BP terms were retained according to the significance threshold defined by the software. The resulting enriched biological processes were subsequently used to functionally annotate the retained treatment-associated WGCNA modules and to facilitate the biological interpretation of the prioritized candidate lncRNAs.

## 5. Conclusions

In conclusion, this study provides a modular RNA-seq framework for identifying stress-responsive lncRNAs that are embedded within biologically coherent co-expression networks. By comparing stress modalities and integrating coding and non-coding transcriptomic layers, our analysis highlights a pronounced non-coding transcriptional response to H_2_O_2_-induced oxidative stress and, to a lesser extent, UV-induced DNA damage, whereas hypoxia and heat stress showed more restricted lncRNA signatures under the applied thresholds. The integration of differential expression, WGCNA, and intra-module lncRNA–mRNA co-expression analysis prioritized network-supported candidate lncRNAs that may contribute to stress-adaptive cellular states. Overall, these findings support the role of lncRNAs as stress-responsive regulators and biomarkers of molecular stress types and provide a methodological foundation for future large-scale studies and experimental validation in stress-related human pathophysiology.

## Data Availability

Data derived from the ASTRA database are available through the ASTRA GitHub repository (https://github.com/Mtsif/astra-db, accessed on 20 June 2026), including dataset metadata, links to the original datasets, and processed files. The complete computational framework used in this study, including analysis code, input files, intermediate results, expected outputs, and documentation required to reproduce the analyses presented in the manuscript, is available through Zenodo (https://doi.org/10.5281/zenodo.21856208).

## Supplementary Materials

The following supporting information can be downloaded at: https://www.mdpi.com/article/10.3390/ijms27167288/s1.

## Abbreviations

The following abbreviations are used in this manuscript:

ASTRA	Atlas of Stress Response Activity
GEO	Gene Expression Omnibus
BH	Benjamini–Hochberg
CPM	counts per million
DE	differentially expressed
DE lncRNA	DE long non-coding RNA
DE mRNA	DE messenger RNA
FDR	false discovery rate
GLM	generalized linear model
GO	Gene Ontology
BP	Biological Process
GRCh38	Genome Reference Consortium Human Build 38
H_2_O_2_	hydrogen peroxide
IQR	interquartile range
kME	module membership
log_2_FC	log_2_ fold change
PCA	principal component analysis
RNA-seq	RNA sequencing
STAR	Spliced Transcripts Alignment to a Reference
TMM	trimmed mean of M-values
TOM	topological overlap matrix
UV	ultraviolet
WGCNA	weighted gene co-expression network analysis
