# Supplementary material for: Integrative RNA-Seq Analysis Reveals Stress Type-Dependent lncRNA-Centered Co-Expression Networks Across Human Cellular Stress Contexts
Source: Int J Mol Sci. 2026 Aug 15;27(16):7288. doi: 10.3390/ijms27167288 (PMC13513128; doi:10.3390/ijms27167288)
Supplement: Supplementary file 1 [file ijms-27-07288-s001.zip › Supplementary_titles.pdf]

Figure S1: Classification of differentially expressed lncRNAs according to stress-type-dependent and shared occurrence across stress modalities;
Figure S2: Number of differentially expressed protein-coding genes identified in each stress modality, separated by direction of regulation;
Figure S3: Recurrence of differentially expressed protein-coding genes (DE-mRNAs) across independent GEO studies and cellular models;
Table S1: Study-level and treatment-level metadata summary of the harmonized ASTRA/GEO stress RNA-seq dataset;
Table S2: Full thresholded DE-lncRNA signatures across stress modalities;
Table S3: Full thresholded DE-mRNA signatures across stress modalities;
Table S4: Gene Ontology Biological Process enrichment results across DE-mRNAs;
Table S5: Differentially expressed lncRNAs embedded within treatment-associated WGCNA modules across stress modalities;
Table S6: Cross-study robustness analysis of prioritized candidate lncRNAs;
Table S7: Cross-study robustness analysis of differentially expressed protein-coding genes across retained modules; Table S8: Gene Ontology Biological Process of retained Modules
